# Effect of Non-Thermal Atmospheric Plasma on Micro-Tensile Bond Strength at Adhesive/Dentin Interface: A Systematic Review

**DOI:** 10.3390/ma14041026

**Published:** 2021-02-22

**Authors:** Mohamed M. Awad, Feras Alhalabi, Abdullah Alshehri, Zaid Aljeaidi, Ali Alrahlah, Mutlu Özcan, Hamdi Hosni Hamama

**Affiliations:** 1 Department of Conservative Dental Sciences, College of Dentistry, Prince Sattam Bin Abdulaziz University, Al-Kharj 11942, Saudi Arabia; f.alhalabi@psau.edu.sa (F.A.); am.alshehri@psau.edu.sa (A.A.); z.aljeaidi@psau.edu.sa (Z.A.); 2 Department of Restorative Dental Science, College of Dentistry, King Saud University, Riyadh 11545, Saudi Arabia; aalrahlah@ksu.edu.sa; 3 Engineer Abdullah Bugshan Research Chair for Dental and Oral Rehabilitation, King Saud University, Riyadh 11545, Saudi Arabia; 4 Division of Dental Biomaterials, Center for Dental and Oral Medicine, University of Zürich, 8032 Zürich, Switzerland; mutluozcan@hotmail.com; 5 Department of Operative Dentistry, Faculty of Dentistry, Mansoura University, Mansoura 35516, Egypt; hamdy@connect.hku.hk

**Keywords:** adhesives, resin–dentin bonding, micro-tensile bond strength, non-thermal atmospheric plasma

## Abstract

Objective: The objective of this review was to evaluate the effect of non-thermal atmospheric plasma (NTAP) on adhesives resin–dentin micro-tensile bond strength (μTBS) in previously published studies. Methods: Electronic search was conducted using the Medline, Cochrane library, and Scopus databases. The included studies were laboratory studies that investigated the effect of NTAP on adhesives μTBS to coronal dentin. Studies that evaluated the effect of NTAP on bond strength to indirect substrates, enamel or root dentin, were excluded. The methodological quality of included studies was assessed. Results: Thirteen studies were included in this systematic review. All the included studies were considered to have a medium risk of bias. NTAP significantly improved μTBS at 24 h or after short-term aging in five studies (38.5%) and both immediate and after long-term aging in 5 studies (38.5%). In two studies (15.4%), NTAP resulted in a short-term material-dependent effect that was not stable after long-term aging. Interestingly, in one study (7.7%), NTAP had a positive effect only in the etch-and-rinse (ER) mode after long-term aging. Conclusion: Within the limitations of this systematic review, NTAP application could enhance resin–dentin μTBS of ER adhesives or universal adhesives (UAs) applied in the ER mode. In the ER mode, the rewetting step after NTAP seems to be unnecessary. Because of the limited information currently available in the literature, further studies are required to evaluate the effect of the NTAP application on self-etch (SE) adhesives or UAs applied in the SE mode.

## 1. Introduction

The stability of the resin–dentin interface affects the clinical performance of resin-based composite (RBC) restorations [1]. Resin–dentin bonding can be achieved by either etch-and-rinse (ER) or self-etch (SE) approaches. In the ER strategy, dentin is demineralized using phosphoric acid followed by the washing and drying steps before adhesive application and infiltration into dentin. In the SE approach, an acidic primer or adhesive is used to simultaneously demineralize and infiltrate dentin, so the washing and drying steps are not required [2]. Current adhesives can be categorized into ER adhesives, SE adhesives or universal adhesives (UAs) which are the latest generation of dental adhesives that can be used in either ER or SE strategy [3]. Irrespective of the adhesive strategy applied, the achievement of a durable resin–dentin bonding is a challenging task [4,5], and it relies mainly on the hybrid layer which is created as a result of infiltration of adhesive monomers into the demineralized dentin [6]. The SE strategy is associated poor resin infiltration can be also noticed within the adhesive interface, which adversely affects the resin–dentin bond durability [7]. Further, the etching of dentin with phosphoric acid can result in the formation of thicker hybrid layers, and longer and more well-defined resin tags [8]. However, adhesives may not completely encapsulate the exposed collagen fibrils of acid-etched dentin [9,10].

Resin–dentin bonding can be affected by dentin wetness prior to adhesives application [11,12]. The wetness of acid-etched dentin is a determinant factor affecting the quality of the hybrid layer and dentin bonding with ER adhesives [13]. Moreover, it can affect the outcome of the adhesive application mode [14]. The bonding performance of some UAs applied in the ER strategy may be affected by dentin wetness [15]. However, recent studies [16,17,18] have shown that this effect is material–dependent for UAs. The drying of acid-etched dentin may be inadequate, resulting in overly wet dentin, or excessive drying can lead to dentin desiccation, which in turn could result in the collapse of collagen fibrils within demineralized dentin and inadequate infiltration of adhesive into inter-tubular dentin [19]. Inadequate drying of acid-etched dentin could also result in the suboptimal replacement of loosely bound water within the collagen matrix of demineralized dentin [20]; this is a major factor that adversely affects the durability of resin–dentin bonding [21], owing to the hydrolytic degeneration of collagen [22]. The effect of several approaches, such as the use of collagen cross-linkers [23] and novel solvents [24] on the resin–dentin bond strength and stabilization of the hybrid layer has been investigated.

Plasma is described as partially ionized gases containing highly reactive particles such as electronically excited atoms, molecules and free radical species [25]. Based on the gas temperature, plasmas can be categorized into two main types: thermal (high temperature) and non-thermal (low-temperature or cold) plasmas (NTAP) [26] which can be used in the biomedical applications. Recently, NTAP has gained substantial attention in the field of the adhesive dentistry for non-destructive surface treatment associated with less chances of technical errors and improvement in the dentin surface energy and wettability [27]. Moreover, NTAP may increase the hydrophilicity of the demineralized dentin surface, which enhances adhesive penetration into spaces around collagen fibrils of acid-etched dentin [28]. A previous narrative review generally discussed the effects of NTAP on resin–dentin bonding [29]: however, few studies on the bond strength were considered. Therefore, the objective of this review to evaluate the effect of NTAP on the bond strength to coronal dentin in light of the currently available literature.

## 2. Methods

### 2.1. Methods

The Preferred Reporting Items Systematic Review and Meta-Analysis (PRISMA) statement guidelines [30] were followed in reporting this systematic review. Considering the participants (P), interventions (I), comparators (C), and outcomes (O), and the (PICO) question formula [24], the research question for this systematic review was as follows: “Can NTAP treatment (I), compared to no NTAP treatment (C), affect adhesives micro-tensile bond strength (μTBS) (O) in case of bonding to coronal dentin (P)?”

### 2.2. Information Sources and Systematic Search

The search keywords “Non thermal plasma” or “cold plasma,” “adhesive,” “bond strength,” and “dentin” or “dental” were used to perform the electronic search in three databases, namely, Medline, Web of Science, and Scopus, in order to identify studies that investigated the effect of NTAP on resin–dentin (μTBS) and that were published between Jan 01, 1990, and Oct 04, 2020, in the English language. In addition, the reference lists of the included studies were searched manually to identify relevant studies. The identified studies were imported into Endnote X7.7 software (Thompson Reuters, Philadelphia, PA, USA) and duplicated items were removed.

### 2.3. Search Strategy

After the duplicated studies were removed, all the remaining identified studies were distributed among the seven authors of this paper. The eligibility criteria were checked independently by each author at the title/abstract level for all articles and at the full-text level for selected articles.

The included studies had to be laboratory studies in which the effect of NTAP on the μTBS of adhesives in the case of bonding to coronal dentin was evaluated and published in the English language. The exclusion criteria were as follows: (1) studies irrelevant with respect to the study question, (2) reviews, (3) clinical studies, and (4) studies that evaluated the effect of NTAP on the bond strength in the case of bonding to dental substrates other than coronal dentin or studies in which adhesives resin–dentin μTBS was not evaluated. For this systematic review, at least six authors were required to agree to the inclusion/exclusion of any study.

### 2.4. Data Extraction and Bias Risk Assessment for the Included Studies

The details of the included studies and their main outcomes in relation to the research question are summarized in Table 1. Moreover, the adhesive procedures applied in each study are detailed in Table 2. The methodological quality of each included study was independently evaluated by the authors according to parameters that are adopted and modified from previous systematic reviews of studies on the in vitro bond strength [31,32]: These parameters are teeth randomization [31,32], teeth free of caries [31,32], blinding of the examiner [31,32], samples with similar dimensions [32], evaluation of the failure mode [32], sample size calculation [31,32], and complete NTAP specifications and application details (working gas, flow rate, power input, application time, and distance of NTAP source). During assessment of each study, if the presence of some of these parameters was identified, the study was said to have a “Yes” for each specific parameter; if the information was not be obtained, the study received a “No.” Studies that reported one or two items were considered to have a high risk of bias, and if they reported three to five of the said parameters, they were considered to have a medium risk of bias; similarly, they were considered to have a low risk of bias if they reported six or seven items.

## 3. Results

### 3.1. Search Results

The electronic search in all databases identified 2287 published articles. After the removal of duplicated studies, the initial screening of the 1169 search results independently performed by the authors at the title/abstract level resulted in the exclusion of 1100 studies because of one or more of the following reasons: irrelevant to research question, review articles, clinical studies, and studies evaluating the effect of NTAP on the implant surface and osteointegration, biofilm and disinfection, and not written in English language.

**Figure 1:**
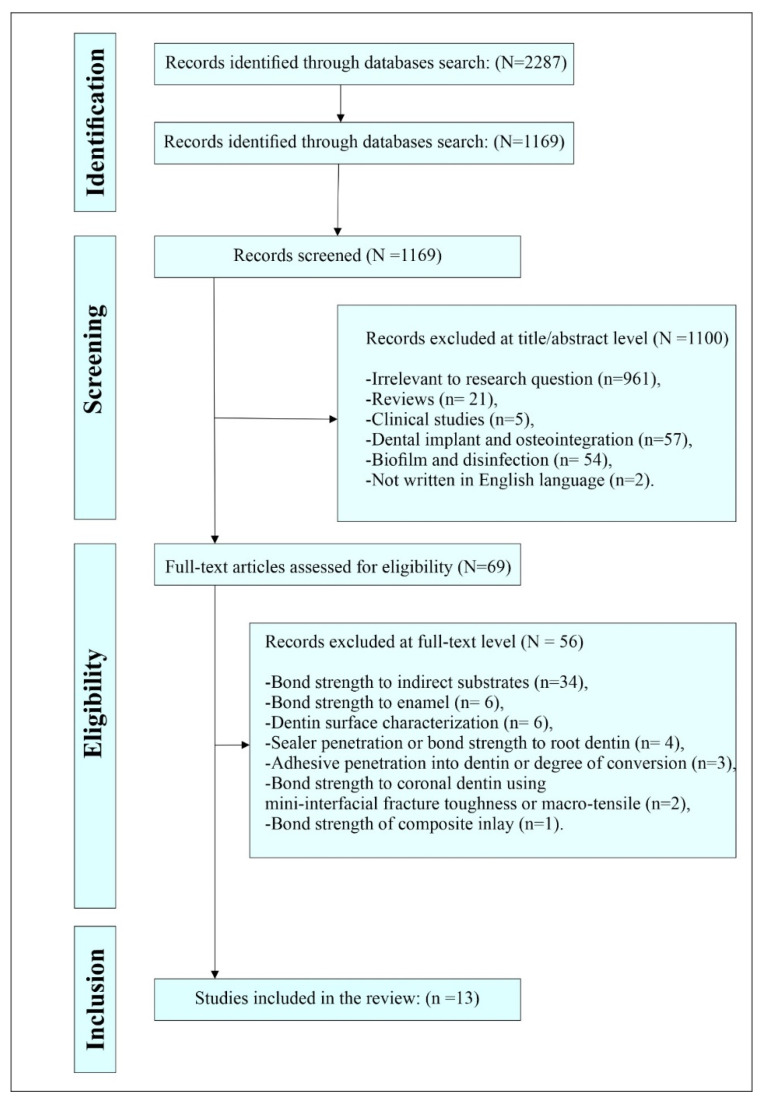
Search flowchart as adapted from the Preferred Reporting Items Systematic Review and Meta-Analysis (PRISMA) statement guidelines.

Sixty-nine studies were assessed at the full-text level for eligibility, and fifty-six studies were excluded. The excluded studies evaluated the effect of NTAP on: (1) bond strength to indirect substrates, (2) bond strength to enamel, (3) dentin surface characterization, (4) sealer penetration or bond strength to root dentin, (5) adhesive penetration into dentin or degree of conversion, (6) bond strength to coronal dentin using mini-interfacial fracture toughness or macro-tensile and (7) bond strength of composite inlay. Finally, thirteen studies satisfied the inclusion criteria and were included for qualitative analysis in this systematic review. The search stages are illustrated in Figure 1.

The included studies are summarized in Table 1, which provides the details regarding NTAP specifications and application, adhesives used, sample size, and aging applied in the μTBS test and the study’s main outcome in relation to the research question. Moreover, the adhesive procedures applied in each included study are detailed in Table 2, which lists details about the status of the bonded substrate, use of phosphoric acid etching, dentin moisture, NTAP function, dentin rewetting, adhesive application, air-drying and light-curing steps, and bonded material.

### 3.2. Descriptive Analysis

In the 13 studies, two NTAP gases (helium in five studies (38.5%) and argon in eight studies (61.5%) were used at a flow rate of 2000 to 5000 sccm and power input of 0.3 to 60 W to evaluate the effect of NTAP on the μTBS of eight adhesives (five ER, two SE, and one MM) bonding to coronal dentin. Further, the dentin was blot-dried in six studies (46.2%) and was wet or moist in two studies (15.4%) before NTAP application, and the dentin moisture not clearly mentioned in five studies (38.5%). The NTAP application durations varied between 5 to 300 s, with 30 s being the most frequently used application time as it was used in 11 studies (86.6%). The distance of the NTAP source varied between 5 and 15 mm. However, it was not clearly mentioned in five studies (38.5%). In four studies (30.8%), the μTBS was evaluated immediately, while artificial aging of samples was performed in nine studies (69.2%). NTAP significantly improved μTBS at 24 h or after short-term aging in five studies (38.5%) and both immediate and after long-term aging in 5 studies (38.5%). In two studies (15.4%), NTAP resulted in a short-term material-dependent effect that was not stable after long-term aging. Interestingly, in one study (7.7%), NTAP had a positive effect only in the ER mode after long-term aging. The dentin substrate was sound in 11 studies (84.6%) sound, and it was treated with 2.5% NaOCl in one study (7.7%). Rewetting of dentin was performed after NTAP application in six (46.2%) studies, not performed in another six (46.2%) studies, while in one study (7.7%), the effect of rewetting after NTAP was evaluated. The adhesive application time and air-drying time were not precisely mentioned in six (46.2%) and two (15.4%) studies, respectively.

Based on the parameters used for the assessment of risk of bias, all the included studies were considered to have a medium risk of bias. The scores of the included studies are presented in Table 3. The included studies scored poorly in terms of two items, namely, sample size calculation and blinding of the examiner.

## 4. Discussion

**Figure 2:**
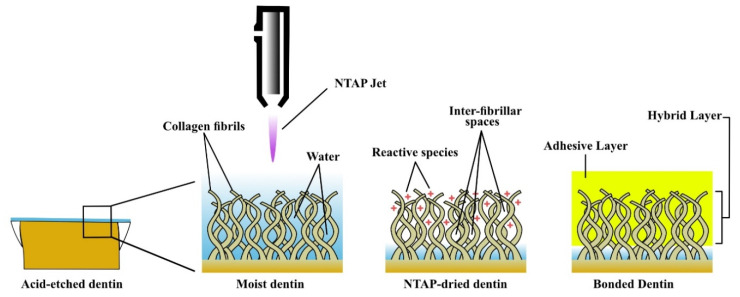
The effect of non-thermal atmospheric plasma (NTAP) on adhesive resin–dentin bonding in etch-and-rinse (ER) mode.

Systematic reviews can help in the healthcare decision-making as they gather, summarize, and evaluate all studies discussing a specific research question [45]. Thus, they serve to identify gaps in the literature and suggest avenues for future studies. [46] PRISMA statement guidelines were followed in reporting this systematic review as it is recommended in reporting dental systematic reviews [45] and the PRISMA endorsement is associated with more thorough reporting compared to other guidelines [47]. Different bond strength tests can be used to evaluate resin–dentin bonding [48]. Only studies in which the μTBS test was applied were included in this systematic review as the μTBS test is a versatile and effective method for evaluating the resin–dentin bond strength [49,50,51], that may correlate with clinical outcomes better than other bond strength tests [52]. NTAP application had a significant positive effect on the resin–dentin μTBS [33,34,35,36,37,38,40,41,42,43,44]. While in two studies [27,39], NTAP application resulted in only a short-term material-dependent positive effect that was not stable after long-term aging. It is noteworthy that the effect of NTAP was more prominent with ER adhesives or UAs applied in the ER mode. In contrast, in the case of SE adhesives or UAs applied in the SE strategy, short- or medium-term μTBS results showed the positive effect of NTAP [40,41], while long-term results indicated no effect [34] or material-dependent effect [27] of NTAP. The treatment of demineralized dentin surfaces with NTAP has been known to increase the penetration of adhesives, resulting in improved adhesion to resin. [28,42,43,44] Upon the qualitative assessment of resin–dentin interfacial morphology using SEM, in the case of the SE mode, no significant difference was detected between NTAP-treated and non-treated groups [27,35,41]. However, in the ER mode, the resin tags formed following NTAP application were longer, well-defined and more abundant compared to non-treated groups [35,37,38,42,43], which was in contrast to the results obtained by Hirata et al. [39] However, the interpretation of adhesive penetration (resin tags) into dentin is controversial. [53] Thus, it cannot be used alone to explain the positive impact of NTAP on resin–dentin μTBS. NTAP might enhance the hybrid layer integrity in two aspects. First, it may have stiffening effect on the hybrid layer, as confirmed by the results of short-term evaluations of the nano-hardness and Young’s modulus [34], and it can apparently inhibit the matrix metalloproteinase (MMPs) enzymatic activity in the ER mode. [35] NTAP application resulted in the formation of a thicker hybrid layer in the ER mode, as observed in SEM assessment [37,43]. In addition, the micro-Raman spectroscopy analysis indicated better penetration of the adhesive resin into the hybrid layer. [33,38] Another possible explanation for the effects of plasma drying on the improved bond strength and its mechanism is that breakdown of interfibrillar bonds, such as hydrogen bonds, might induce structural changes in exposed collagen fibers (Figure 2), thereby preventing collapse of the collagen networks under dry conditions [44,54]. NTAP enhanced the resin infiltration into the collagen network, and this could have improved the immediate μTBS and also might have protected the collagen structure in addition to inhibiting the MMPs enzymes [35]: thus the durability of bonding is improved [33]. As it has been reported that partially encapsulated or exposed dentin collagen fibrils at the hybrid layer [9,10] and susceptible to hydrolytic degradation over time [55]. Previous studies have shown that wet bonding can deteriorate the resin–dentin interface [56], resulting in its degradation over time. [57] However, this effect is material-dependent for current UAs [18]. While NTAP application was followed by rewetting of the NTAP-treated dentin surface to achieve wet-bonding in six studies (46.2%), this step (rewetting) was omitted in another six studies (46.2%). Thus, it seems that there is no consensus on dentin rewetting after NTAP treatment. Dentin rewetting after NTAP application may result in a significant reduction in the charges, while covalent modifications of the collagen fibrils will endure for the adhesive application [44]. In addition, rewetting of the NTAP-treated dentin surface may result in total or partial reduction in its wettability [34,41]. The results of one study [38] that evaluated the effect of rewetting after NTAP drying revealed that NTAP drying alone can result in a higher bond strength compared to wet-bonding (rewetting). This was explained by the maintenance of the collagen network despite the water loss, which resulted in the uniform and homogeneous adhesive–dentin interface [38]. Moreover, in multiple studies, the omission of the rewetting step after NTAP application did not seem to prevent the enhancement of resin–dentin μTBS [38]. Although NTAP may significantly increase the dentin wettability [27,34,41,58,59] due to elimination of the carbon-containing materials or organic substances from the dentin surface, as confirmed by the XPS analysis results [58,59], its effect on adhesives applied in the SE mode was less clear compared to the effect in the ER mode. This can be explained by the fact that NTAP causes no discernable topographic changes (roughness) to dentin [35,59]. Moreover, in the SE mode, there are no acid-etching and washing steps that require drying with air or NTAP, and adhesives simultaneously demineralize and infiltrate the dentin while the collagen fibrils are not exposed. The included studies presented a medium risk of bias. Despite their use in previous studies, the criteria used in the assessment of the risk of bias seem to be rather general and not topic specific. Previous systematic reviews [60] used topic-specific criteria in order to be in accordance with the research question. Similarly, in this study, NTAP specifications were of the aspects used to assess the risk of bias for the included studies. Thus, the adhesive application protocol—including the application time [61] and method [62] in addition to adhesive air-drying [60] could significantly affect the bond strength achieved. In studies on the bond strength, more details on the adhesives application protocol should be precisely described (instead of simply mentioning that application was performed “according to manufacturer’s instructions”). This seems to be essential as such instructions may not be clearly described by some manufacturers. Helium- and argon-generated NTAP can result in different amounts of reactive species [63], thus studies comparing the effect of different NTAP gases is recommended. Studies evaluating the effect of NTAP on the chemical reaction or nano-layering between adhesives and dentin are also recommended. This systematic review is limited by the lack of quantitative evaluation of evidence by statistical analysis achieved through meta-analysis [64] that could not be conducted due to methodological heterogeneity among included studies, particularly in terms of the NTAP specifications (gas type, flow rate, power, application time, and distance of NTAP source) and aging conditions. Moreover, the small number (8) of adhesives tested in the included studies is one of the limitations of this systematic review.

## 5. Conclusions

Within the limitations of this systematic review, NTAP application could enhance resin–dentin μTBS of ER adhesives or UAs applied in the ER mode. The NTAP effect could be a result of the enhancement in the quality of the hybrid layer formed [33,37,38,43] and the inhibition of the MMPs enzymatic activity [35]. In the ER mode, NTAP can be considered an effective drying method of acid-etched dentin, and the rewetting step after NTAP seems to be unnecessary. Because of the limited information currently available in the literature, further studies are required to evaluate the effect of the NTAP application on SE adhesives or UAs applied in the SE mode.

## Figures and Tables

**Table 1:** Data extraction items from the studies included in the review.

	Study	NTAP Specifications		NTAP Application		Adhesive	μTBS		Main Outcome
		Working Gas	Power Input	Time	Distance	-	Sample Size	Aging	-
1.	Han et al., 2019 [33]	Helium Flow rate (FR): 5000 sccm	3 W	30 s	5 mm	Adper Single Bond 2 Adhesive, 3M ESPE; St Paul, MN, USA.	(*n* = 6) Tooth	24 h 10,000 Thermocycling (TC)	NTAP treatment enhanced resin-dentin μTBS, at 24 h and after thermocycling.
2.	Ayres et al., 2018 [34]	Argon FR:5000 sccm	N/A	10, 30 s	10 mm	Scotchbond Universal, 3M ESPE; St Paul, MN, USA.	(*n* = 8) Tooth	One week Two years water storage (WS)	NTAP treatment (30 s) enhanced resin-dentin μTBS for ER mode after aging. NTAP treatment had no significant effect on resin–dentin μTBS for SE mode, at 1 week and after aging.
3.	Ayres et al., 2018 [35]	Argon FR:5000 sccm	N/A	10, 30 s	10 mm	Scotchbond Universal 3M ESPE; St Paul, MN, USA.	(*n* = 8) Tooth	24 h One year WS One year simulated pulpal pressure	NTAP treatment had no significant effect on resin–dentin μTBS for ER or SE modes, at 24 h or after aging using direct water storage. NTAP treatment enhanced resin-dentin μTBS for ER or SE modes, after aging for 1 year under simulated pulpal pressure.
4.	Zhu et al., 2018 [36]	Helium FR:2000 sccm (Conventional) FR:4000 sccm (Modified)	Conventional: V*_pp_* = 67 kV, Frequency = 13.56 MHz Modified: 15 W, Frequency = 13.56 MHz	5, 10 s (conventional) 15, 30, 45, 60 s (modified)	10 mm	Adper Single Bond Plus, 3M ESPE; St. Paul, MN, USA.	(*n* = 4) Tooth	24 h 50,000 TC	Modified NTAP drying enhanced resin-dentin μTBS, at 24 h and after aging. Conventional NTAP (5 s) enhanced resin-dentin μTBS, while dentin treatment for 10 s had a negative effect on μTBS, at 24 h and after aging.
5.	Zhu et al., 2018 [37]	Helium FR:4000 sccm	15 W Frequency = 13.56 MHz	N/A	N/A	Adper Single Bond Plus, 3M ESPE; St. Paul, MN, USA.	(*n* = 4) Tooth	24 h One year (chloramine solution storage)	NTAP drying enhanced resin-dentin μTBS, at 24 h and after aging. The highest μTBS was obtained at 30–45 s.
6.	Kim et al., 2016 [38]	Helium FR:2000 sccm	0.3 W	20 s	5 mm	Adper Single Bond 2, 3M ESPE; St Paul, MN, USA.	(*n* = 24) Hour-glass slabs	24 h	NTAP drying enhanced resin-dentin μTBS. The rewetting after NTAP negatively affected the resin-dentin μTBS.
7.	Hirata et al., 2016 [39]	Argon FR:5000 sccm	8 W Frequency:1.1 MHz, 2 to 6 kV peak-to-peak	30 s	15 mm	Optibond FL, Kerr, Orange, CA, USA. XP Bond, Dentsply De Trey; Konstanz, Germany.	(*n* = 6) Tooth	One week WS One year WS	NTAP treatment before acid etching had no significant effect on resin-dentin μTBS of two-step ER adhesive, at 24 h or after aging. NTAP drying enhanced resin-dentin μTBS of two-step ER adhesive, at 24 h. However, this effect was not stable after aging.
8.	Abreu et al., [40] 2016	Argon FR:N/A	60 W	15, 30, 45 s	N/A	Clearfil SE Bond, Kuraray Noritake Dental; Kurashiki, Japan.	(*n* = 5) Tooth	48 h WS	NTAP treatment (30 s) enhanced resin-dentin μTBS, promoting chemical changes in the dentin structure.
9.	Hirata et al., 2015 [27]	Argon FR:5000 sccm	8 W Frequency:1.1 MHz, 2 to 6 kV peak-to-peak	30 s	15 mm	Clearfil SE Bond, Kuraray Noritake Dental; Kurashiki, Japan. Scotchbond Universal, 3M ESPE; St Paul, MN, USA.	(*n* = 6) Tooth	One year WS	NTAP treatment enhanced resin-dentin μTBS for the universal adhesive, at 24 h. However, this positive effect was not stable after aging. NTAP treatment had no significant effect on resin-dentin μTBS for the two-step SE adhesive, at 24 h or after aging
10.	Dong et al., 2015 [41]	Argon FR:3000 sccm	2–3 W	30 s	N/A	OptiBond All-In-One, Kerr; Romulus, MI, USA.	(*n* = 8) Tooth	24 h WS 60 days WS	NTAP treatment enhanced resin-dentin μTBS, at 24 h and 60 days.
11.	Han et al., 2014 [42]	Helium FR:2000 sccm	Conventional: 21.6 kW h Pulsed: 1.1 kW h	30 s	5 mm	Scotchbond Multi-Purpose Plus adhesive system, 3M ESPE; St Paul, MN, USA.	(*n* = 20) Hour-glass slabs	24 h 5000 TC	Both types of NTAP drying enhanced resin-dentin μTBS at 24 h and after thermocycling.
12.	Dong et al., 2013 [43]	Argon FR:3000 sccm	2–3 W	30 s	N/A	Adper Single Bond Plus, 3M ESPE; St. Paul, MN, USA.	(*n* = 8) Tooth	24 h	NTAP drying enhanced resin-dentin μTBS.
13.	Ritts et al., 2010 [44]	Argon FR:2500 sccm	5 W	30, 100, 300 s	N/A	Adaper Single bond plus, 3M ESPE; St Paul, MN, USA.	N/A	24 h	NTAP drying (30 s) enhanced resin-dentin μTBS. Prolonged plasma treatment could lead to a weak interface and deteriorated dentin micromechanical properties.

**Table 2:** Adhesive procedures applied in studies included in the review.

	Study		Adhesive Procedures							
		Substrate (Dentin)	Acid Etching/Time	Dentin Moisture	NTAP Function	Rewetting	Adhesive			Bonded Material
							Application	Air-Drying	Light-Curing	
1.	Han et al. 2019 [33]	NA	35% H_3_PO_4_/	Wet	Drying of demineralized dentin	No	N/A	Gently air-dried	10 s	RBC
2.	Ayres et al., 2018 [34]	Sound	34% H_3_PO_4_/(ER), No (SE)	N/A	Dentin surface treatment Drying of demineralized dentin	No	Manufacturer’s Instructions	Manufacturer’s Instructions	Manufacturer’s Instructions	RBC
3.	Ayres et al., 2018 [35]	Sound	34% H_3_PO_4_/(ER), No (SE)	Blot-dried	Dentin surface treatment Drying of demineralized dentin	No	Manufacturer’s Instructions	Manufacturer’s Instructions	10 s	RBC
4.	Zhu et al., 2018 [36]	Sound	32% H_3_PO_4_	Blot-dried	Drying of demineralized dentin	Yes	N/A	Air-thined	15 s	RBC
5.	Zhu et al., 2018 [37]	Sound	32% H_3_PO_4_	Blot-dried	Drying of demineralized dentin	Yes	N/A	Air-thined	15 s	RBC
6.	Kim et al., 2016 [38]	Sound	35% H_3_PO_4_	N/A	Drying of demineralized dentin	Yes/No	N/A	Gently air-dried	10 s	RBC
7.	Hirata et al., 2016 [39]	Sound	35% H_3_PO_4_	N/A	Drying of demineralized dentin	No	Manufacturer’s Instructions	Manufacturer’s Instructions	Manufacturer’s Instructions	RBC
8.	Abreu et al., 2016 [40]	2.5% NaOCl	No	N/A	Dentin surface treatment	No	Manufacturer’s Instructions	Manufacturer’s Instructions	Manufacturer’s Instructions	RBC
9.	Hirata et al., 2015 [27]	Sound	No	N/A	Dentin surface treatment	No	Manufacturer’s Instructions	Manufacturer’s Instructions	Manufacturer’s Instructions	RBC
10.	Dong et al., 2015 [41]	Sound	No	Moist	Dentin surface treatment	Yes	Manufacturer’s Instructions	Manufacturer’s Instructions	10 s	RBC
11.	Han et al., 2014 [42]	Sound	35% H_3_PO_4_	Blot-dried	Drying of demineralized dentin	Yes	Manufacturer’s Instructions	Manufacturer’s Instructions	10 s	RBC
12.	Dong et al., 2013 [43]	Sound	37% H_3_PO_4_	Blot-dried	Drying of demineralized dentin	Yes	N/A	N/A	10 s	RBC
13.	Ritts et al., 2010 [44]	Sound	37% H_3_PO_4_	Blot-dried	Drying of demineralized dentin	Yes	N/A	N/A	10 s	RBC

**Table 3:** Assessment of risk of bias for the included studies.

	Study	Randomization	Caries Free	Similar Dimensions Samples	Sample Size Calculation	Blinding of Examiner	Failure Mode	NTAP Specifications and Application	Risk of Bias
1.	Han et al., 2019 [33]	Yes	No	Yes	No	No	Yes	Yes	Medium
2.	Ayres et al., 2018 [34]	No	Yes	Yes	No	No	Yes	Yes	Medium
3.	Ayres et al., 2018 [35]	Yes	Yes	Yes	No	No	Yes	Yes	Medium
4.	Zhu et al., 2018 [36]	Yes	Yes	Yes	No	No	No	Yes	Medium
5.	Zhu et al., 2018 [37]	Yes	Yes	Yes	No	No	No	No	Medium
6.	Kim et al., 2016 [38]	Yes	Yes	Yes	No	No	Yes	Yes	Medium
7.	Hirata et al., 2016 [39]	Yes	Yes	Yes	No	No	Yes	Yes	Medium
8.	Abreu et al., 2016 [40]	No	No	Yes	Yes	No	Yes	No	Medium
9.	Hirata et al., 2015 [27]	Yes	Yes	Yes	No	No	Yes	Yes	Medium
10.	Dong et al., 2015 [41]	Yes	Yes	Yes	No	No	Yes	No	Medium
11.	Han et al., 2014 [42]	Yes	Yes	Yes	No	No	Yes	Yes	Medium
12.	Dong et al., 2013 [43]	No	Yes	Yes	No	No	Yes	No	Medium
13.	Ritts et al., 2010 [44]	No	Yes	Yes	No	No	Yes	No	Medium

## Data Availability

The data presented in this study are available on request from the corresponding author.
